# IL-6–174 G/C transversion might decrease male infertility risk: A case-control study

**DOI:** 10.18502/ijrm.v22i12.18067

**Published:** 2025-01-31

**Authors:** Tayyebeh Zamani-Badi, Mohammad Karimian, Javad Amini Mahabadi, Atieh Rafatmanesh, Hossein Nikzad

**Affiliations:** ^1^Anatomical Sciences Research Center, Institute for Basic Sciences, Kashan University of Medical Sciences, Kashan, Iran.; ^2^Department of Molecular and Cell Biology, Faculty of Basic Sciences, University of Mazandaran, Babolsar, Iran.; ^3^Gametogenesis Research Center, Kashan University of Medical Sciences, Kashan, Iran.

**Keywords:** Polymorphism, Genetic, Interleukin-6, Spermatogenesis, Male infertility.

## Abstract

**Background:**

Genetic predispositions have been identified as important factors in male infertility. Among the many genes related to male reproductive function, interleukin 6 (IL-6) has emerged as a key player. Despite the growing recognition of genetic factors in male infertility, the specific association between the IL-6–174 G/C genetic polymorphism and male infertility remains an area that needs further investigation.

**Objective:**

This investigation explores the correlation between the IL-6–174 G/C transversion and male infertility.

**Materials and Methods:**

In a case-control study, a total of 314 men who referred to the Kashan Infertility Center, Shahid Beheshti hospital, Kashan, Iran, were enrolled for IL-6–174 G/C polymorphism analysis. The study comprised 163 infertile participants as the case group and 151 fertile men as the control group. Following the screening, 2 ml of whole blood was collected from each participant. Cases were categorized into 3 subgroups based on World Health Organization criteria: (i) nonobstructive azoospermia (n = 42), (ii) oligozoospermia (n = 61), and (iii) asthenozoospermia (n = 60). After DNA extraction, genotypes of the samples at the -174 G/C (rs1800795) locus were determined using the polymerase chain reaction-restriction fragment length polymorphism method.

**Results:**

Our genetic investigation demonstrated a significant association between the GC genotype and male infertility. Furthermore, a correlation was observed between the heterozygous GC genotype and reduced risk of oligozoospermia and asthenospermia. Additionally, the C allele was correlated with a decreased risk of infertility and specific subgroups such as oligozoospermia and asthenospermia.

**Conclusion:**

Our findings suggest that the IL-6–174 G/C transversion could potentially serve as a protective genetic factor against male infertility.

## 1. Introduction

Infertility is a complex issue influenced by various factors, including anatomical, environmental, lifestyle, physiological, and genetic components. Male infertility is a prevalent concern affecting around 5% of men (1).

While there are several causes of male infertility, including post-testicular obstruction, varicocele, anti-sperm antibody production, and hormonal imbalances, genetic factors are regarded as one of the primary contributing factors (2). Studies have demonstrated that genetic factors such as aneuploidies, Y chromosome microdeletions, mitochondrial DNA abnormalities, point mutations, and variations in key genes can heighten the risk of male infertility (3). Our prior research has also established associations between genetic polymorphisms in certain cytokine gene families, such as interleukin (IL)-1α, IL-1β, IL-1RA, and susceptibility to male infertility (4, 5).

Interleukin 6 (IL-6) is a versatile cytokine found in seminal fluid, typically produced by various cell types, including Sertoli and germ cells (6). Consequently, it plays a regulatory role in the growth, differentiation, and function of sperm. IL-6 levels have been found to correlate with the secretory activity of Sertoli cells and the count of leukocytes in seminal fluid. Moreover, IL-6 is a key marker for detecting inflammation in seminal fluid (7, 8).

Several single nucleotide polymorphisms (SNPs) within the IL-6 promoter region such as -174G > C, -572G > C, -597G > A, -634C > G, -1363 G > T, and -2954G > C have been extensively studied in relation to various diseases, such as deep vein thrombosis, type 2 diabetes mellitus, and cardiovascular conditions (9–12). Particularly, the -174G > C polymorphism has shown an association with male infertility (13). This polymorphism is an upstream genetic variation located at the promoter region. The mutated allele in this polymorphism is C, serving as a replacement for the wild-type allele G (14). Based on their position in gene regulatory regions, these polymorphisms may cause changes in gene expression (15).

The objective of our study was to investigate the potential link between the IL-6–174G/C SNP and the risk of male infertility.

## 2. Materials and Methods

### Participants with inclusion and exclusion criteria

A case-control study was carried out involving a total of 314 participants, comprising 163 cases and 151 controls. The sample collection was performed between August 2015 and November 2017. Among a total of 314 participants recruited for this investigation, 163 cases were selected from individuals seeking assistance at the Kashan Infertility Center, specifically Shahid Beheshti hospital in Kashan, Iran.

Infertile men included in this study had no history of systemic or other identified genetic diseases, as determined through interviews. Those with conditions such as orchitis, maldescensus of the testis, varicocele, vas deferens obstruction, immune or infectious abnormalities, drug abuse, diabetes mellitus, abnormal hormone profiles (luteinizing hormone, follicle-stimulating hormone, and testosterone), abnormal karyotype, and Y-chromosome microdeletions were excluded. Based on spermiogram results, infertile men were classified into subgroups of oligozoospermia (n = 61), asthenozoospermia (n = 60), and nonobstructive azoospermia (NOA; n = 42). The control group consisted of fertile men with normal sperm parameters, randomly selected, who had at least one offspring without assisted reproductive technologies, and were referred to the same clinic. Additionally, men with any known genetic or familial diseases were excluded from the control group. The demographic and clinical features of cases and controls are summarized in table I. After completing the screening process, a 2 ml sample of whole blood was collected from each participant.

### Sample size

Based on a similar study, and using the online server, for comparing a ratio or outcome between 2 groups, considering a significance level (α) of 0.05, study power of 80%, and the maximum observed mutant allele percentage in patients as 40.4% and in healthy individuals as 20%, the sample size for each group is estimated to be 78 individuals (13, 16). However, due to our access, we were able to collect a larger sample size.

### DNA extraction and genotype analysis

Genomic DNA was extracted from blood samples using a commercially available DNA extraction kit (Bioneer Co., Daejeon, Korea). The genotyping of IL-6 polymorphism was done using the polymerase chain reaction (PCR)-restriction fragment length polymorphism (RFLP) method. For this, specific primers were designed in the vicinity of the polymorphic region using Oligo7 software. The sequences of the forward and reverse primers were as follows:

Forward primer (F): 5 ' -GAAGAGTGGTTCTGCTTCT TAG-3 ' and reverse primer (R): 5 ' -TTTGATAAATC TTTGTTGGAGG-3 ' .

**Figure 1:**
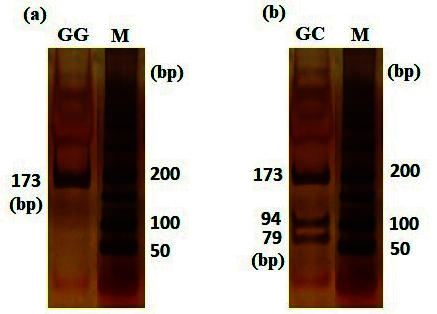
PCR-RFLP results. The (a) and (b) are samples with GG and GC genotypes, respectively, (M: 50 bp DNA leader). To verify PCR-RFLP results, some samples were randomly sequenced.

PCR was conducted in a total volume of 20 μ l containing 10 μ l premix (CinnaGen, Iran), 0.5 μ M of forward and reverse primers, and 2 μ l (30 ng) template DNA. The PCR was performed in a Peqlab peqSTAR thermal cycler with the following program: 94 C for 5 min, 94 C for 45 sec, 53 C for 45 sec, 72 C for 45 sec, 35 cycles, and final 72 C for 5 min. Subsequently, 5 μ l of the PCR products were subjected to digestion using 0.8 units of Hin1II restriction enzyme (#ER1831-Thermo Scientific, Lot: 00471907) with an incubation period of 16 hr at 37 C. The enzyme was subsequently inactivated through incubation at 65 C for 20 min. The digested fragments were separated by electrophoresis on the 8% polyacrylamide gel and visualized using DNA Green Viewer (CinnaGen CO.). Following electrophoresis, 3 distinct genotypes were identified based on their differing sizes: GG (173bp), GC (173, 79, 94), and CC (94, 79) (Figure 1).

**Table 1:** Demographic and clinical parameters of the subjects

**Variables**	**Controls (n = 151)**	**Cases (n = 163)**	**P** ***-*** **value**
Age (yr)*	33.72 ± 5.28 (21–45, 34, 6)	33.53 ± 5.00 (21–44, 34, 8)	0.75
**Smoking (Y/N)^#^**	53/98	68/95	0.23
BMI (kg/m^2^)*	24.42 ± 2.31 (20–30, 25, 3)	24.45 ± 2.60 (19–30, 25, 4)	0.90
Seminal volume (mL)*	3.34 ± 3.34 (1.5–4.5, 3.5, 1)	3.19 ± 0.95 (1.4–4.6, 3.5, 1.7)	0.11
Sperm count ( × 10^6^/mL)*	59.83 ± 11.19 (34–80, 60, 20)	17.21 ± 17.59 (0–70, 10, 30)	< 0.001
Motility (% motile)*	56.18 ± 10.65 (40–80, 60, 20)	23.59 ± 20.18 (0–65, 16, 42)	< 0.001
Morphology (% normal)*	53.00 ± 11.31 (20–69, 55, 16)	31.96 ± 22.58 (0–65, 44, 55)	< 0.001
Data presented as mean ± SD (range, median, interquartile range). BMI: Body mass index. *Quantitative data were analyzed using the *t* test, while #qualitative data were analyzed using the Chi-square test. Significant differences are indicated in bold font			

### Ethical Considerations

Sample collection was conducted after obtaining informed consent from the participants. This study received ethical approval from Kashan University of Medical Sciences, Kashan, Iran (Code: IR.KAUMS.REC.1394.58).

### Statistical Analysis

Statistical analysis for the case-control study was conducted using Statistical Package for the Social Sciences (SPSS) 22 software for Windows (SPSS Inc., Chicago, USA). To assess the normal distribution of continuous variables, the Kolmogorov-Smirnov test was employed for adjustment. Numerical variables were analyzed with *t* test. The Hardy-Weinberg equilibrium of observed genotype frequencies was determined using a goodness-of-fit Chi-square test. Associations between categorical variables, including genotype frequencies and odds ratios, were evaluated using the Chi-square test. A significance level of p < 0.05 was considered statistically significant.

## 3. Results

The data demonstrated a significant association between the heterozygous genotype GC and a reduction in the risk of male infertility. Furthermore, the data indicated a meaningful correlation between the GC genotype and a decreased risk of oligozoospermia, and asthenospermia. Additionally, a significant association was observed in a dominant genetic model (GC + CC vs. GG) between the IL-6–174 G/C polymorphism and a decreased risk in male infertility, extending to the subgroups of oligozoospermia and asthenospermia (Table II). The allelic analysis revealed that the C allele was associated with a decreased risk of male infertility. Furthermore, a significant association was identified between the C allele and a reduced risk of oligozoospermia and asthenospermia (Table II). Due to the rarity of the CC genotype in both infertile and healthy individuals (0 frequency), genetic association analysis was not performed, as it requires larger sample sizes for such analyses.

**Table 2:** Allele and genotype distribution for IL-6174 G/C

		**Groups**					**OR (95% CI)**				**P-value**			
		**Control (n = 151)**	**All cases (n = 163)**	**Oligo (n = 61)**	**Astheno (n = 60)**	**NOA (n = 42)**	**Total**	**Oligo**	**Astheno**	**NOA**	**Total**	**Oligo**	**Astheno**	**Azo**
**Genotype**														
	**GG**	71 (47.02)	112 (68.71)	42 (68.85)	43 (71.67)	27 (64.29)	-	-	-	-	-	-	-	-
	**GC**	80 (52.98)	51 (31.29)	19 (31.15)	17 (28.33)	15 (35.71)	0.40 (0.26–0.64)	0.40 (0.21–0.75)	0.35 (0.18–0.67)	0.49 (0.24–1.00)	> 0.001	> 0.01	> 0.01	0.05
	**CC**	0 (00.00)	0 (00.00)	0 (00.00)	0 (00.00)	0 (00.00)	0.64 (0.01–32.39)	1.68 (0.03–86.36)	1.64 (0.03–84.35)	2.60 (0.05–134.30)	0.82	0.63	0.80	0.63
	**GC+CC**	80 (52.98)	51 (31.29)	19 (31.15)	17 (28.33)	15 (35.71)	0.40 (0.26–0.64)	0.40 (0.21–0.75)	0.35 (0.18–0.67)	0.49 (0.24–1.00)	> 0.001	> 0.01	> 0.01	0.05
**Allele**														
	**G**	222 (73.51)	275 (84.36)	103 (84.43)	103 (85.83)	69 (82.14)	-	-	-	-	-	-	-	-
	**C**	80 (26.49)	51 (15.64)	19 (15.57)	17 (14.17)	15 (17.86)	0.51 (0.35–0.76)	0.51 (0.29–0.89)	0.46 (0.26–0.81)	0.60 (0.33–1.11)	> 0.001	0.02	> 0.01	0.12
Data presented as n (%). A binary logistic regression was used to estimate OR with a 95% CI. OR: Odds ratio, CI: Confidence interval, Oligo: Oligozoospermia, Astheno: Asthenospermia, NOA: Nonobstructive azoospermia, Azo: Azoospermia. Significant differences between the case and control groups are bolded														

## 4. Discussion 

In this research, we investigated the association between IL-6–174 G/C transversion and male infertility within a specific Iranian sub-population located in Kashan, Iran. This case-control study aimed to shed light on whether this genetic transversion could potentially change the risk of infertility. Our findings suggested a notable association between the GC genotype and decreased risk of male infertility, which extended to subgroups, including oligozoospermia and asthenospermia. Furthermore, individuals carrying the C allele exhibited a similar association with male infertility across these subgroups. Allelic analysis indicated that the C allele itself is associated with the decreased risk of infertility, and a significant association was observed regarding the reduced risk of oligozoospermia and asthenospermia.

**Figure 2:**
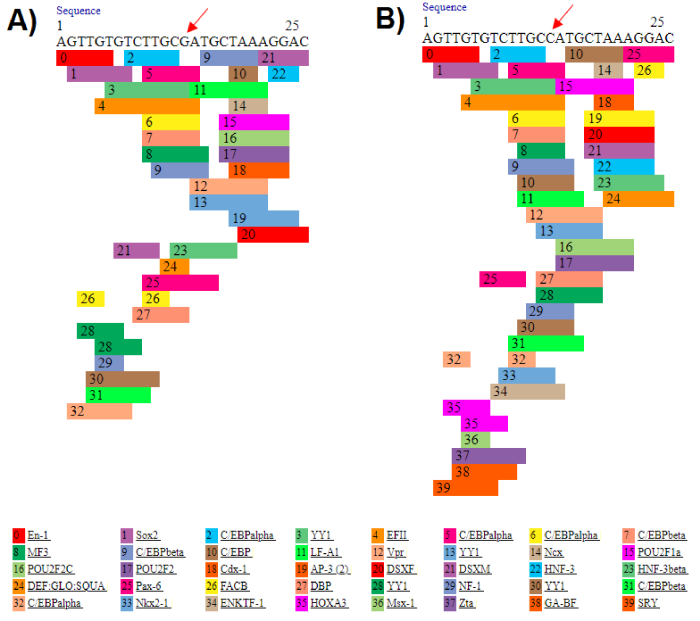
Prediction of transcription factor binding sites upstream of the *IL-6* gene for G and C alleles of the rs1800795 polymorphism. The PROMO web server predicts distinct transcription factors binding to the DNA sequence for genotypes G (A) and C (B). The polymorphic site is indicated by red arrowhead.

Sometimes, mutant alleles can improve survival, increase biological efficiency, and adapt to the environment (17, 18). Numerous studies have indicated that mutant allele of SNPs are protective (19, 20). We stated in our article that the C allele is a protective factor against infertility, but this does not mean that the G allele is the cause of infertility. All genetic polymorphisms and mutations are not necessarily risk factors and can have positive effects on the corresponding protein or even RNA. For example, Mobasseri et al. reported that the mutant allele of rs2234693 polymorphism can be a protective factor against male infertility with a positive effect on RNA stability (21). Given that the polymorphism studied is located upstream of the gene, it may affect transcription factor binding sites. The eukaryotic promoter database indicated that the IL-6–174 G/C polymorphism is positioned close to and upstream of the promoter, and the PROMO server showed that the C variant creates additional binding sites for transcription factors for IL-6 (Figure 2), potentially playing an effective role in the gene regulation. Therefore, this may explain the protective role of the IL-6–174 G/C polymorphism against male infertility.

A previous study conducted in the Uttar Pradesh population of North India also demonstrated an association between the IL-6–174 G/C transversion and male infertility. Their subgroup analysis revealed higher levels of apoptosis and necrosis in individuals with oligozoospermia and asthenospermia compared to the control group. Additionally, they found associations with increased levels of reactive oxygen species (ROS), decreased testosterone and luteinizing hormone, and increased prolactin and follicle-stimulating hormone in the infertile group (13).

Male infertility is a complex, multifactorial condition that poses a significant global public health challenge, with a substantial percentage of affected men experiencing conditions such as azoospermia, asthenozoospermia, and oligospermia. An array of factors, including anatomical, environmental, and genetic factors, contribute to male infertility (22).

The process of spermatogenesis, which is vital for producing mature sperm, is influenced by various cytokines. Elevated levels of IL-6 have been associated with disruptions in spermatogenesis, potentially contributing to infertility (23). Notably, there is an inverse relationship between cholesterol levels and IL-6, with cholesterol playing a role in male gonad parameters like Sertoli cell function, germ cell differentiation, and steroidogenesis (24). Any alteration affecting IL-6 integrity can impact reproductive function, directly or indirectly, and is correlated with sperm lipid peroxidation (25).

Among genetic factors, polymorphisms in cytokine genes can alter their structure, function, or expression levels. Polymorphisms in the *IL-6* gene have the potential to elevate cytokine levels and induce the generation of ROS, which can have detrimental effects on spermatogenesis (13). Roughly, 30–80% of ROS levels can lead to damage in spermatozoa. Elevated ROS levels can damage sperm membranes, DNA, and proteins, resulting in abnormalities in sperm count, motility, and structural integrity (26). Consequently, these disruptions can manifest as various phenotypes, including azoospermia, oligozoospermia, and asthenozoospermia (27).

In many cases of autosomal recessive traits, individuals who are homozygous for mutated allele experience reduced fitness compared to those who are heterozygous or homozygous for the normal allele. However, there are certain disorders where environmental conditions lead to higher fitness in heterozygotes than in either homozygous genotype. This phenomenon is known as heterozygote advantage. It is significant because even a slight advantage in heterozygotes can lead to an increase in the frequency of the mutated allele within the population, even if the mutation significantly reduces fitness in homozygotes. Instances where natural selection simultaneously works to maintain a harmful allele in the gene pool while also working to eliminate it are called balanced polymorphisms (28). The inheritance pattern of SNPs follows the general principles of Mendelian inheritance, with some subtle differences, and in some accepted models including codominant, dominant-recessive, incomplete dominance, overdominance, and additive. Generally, the inheritance pattern of SNPs can vary depending on the specific SNP and its interaction with other genetic and environmental factors. Additionally, SNPs can exhibit different inheritance patterns in populations or different ethnic groups (29, 30).

For examining only one gene as well as one polymorphism, this preliminary study serves as an initial investigation, providing data that will guide future, more thorough research aimed at drawing clinical conclusions regarding whether this genetic variation can serve as a biomarker indicating susceptibility to male infertility. Considering that investigating the effects of genetic polymorphisms through experimental methods such as in vitro and in vivo approaches is costly and time-consuming, computational methods can be a suitable approach for studying the effects of polymorphisms (15). Given that the polymorphism under study, rs1800795, is an upstream variant, its effects on gene function, particularly on gene expression, are suggested to be explored using bioinformatics tools.

However, it is essential to acknowledge the limitations of our study, particularly regarding gene-gene and gene-environment interactions that were not explored. Considering the results of our study and the absence of the homozygous CC genotype in the entire population under study (both in the case and control groups), we were unable to statistically analyze the behavior of this genotype in male infertility, precisely. With the low frequency of the homozygous CC genotype in the population, obtaining a larger sample size and accessing individuals with the homozygous CC genotype will result in more accurate results, regarding this genotype and its association with male infertility. Additionally, obtaining more precise data would necessitate larger study groups of both infertile and control subjects from diverse ethnic populations, as geographical regions can influence study outcomes. Moreover, exploring additional polymorphisms in *IL-6* gene and other inflammatory genes in fertile and infertile men could contribute to obtaining more precise conclusions.

## 5. Conclusion

Our study suggests that the IL-6–174 G/C polymorphism may serve as potential protective factor against male infertility. Nevertheless, future comprehensive studies are required to further elucidate the role of this genetic factor in male infertility.

##  Data Availability

Data supporting the findings of this study are available upon reasonable request from the corresponding author.

##  Author Contributions

H. Nikzad, and M. Karimian designed the study and conducted the research. T. Zamani-Badi, J. Amini Mahabadi, and A. Rafatmanesh evaluated and analyzed the results of the study. Further, M. Karimian, H. Nikzad, T. Zamani-Badi, J. Amini Mahabadi, and A. Rafatmanesh reviewed the article. All authors approved the final manuscript and take responsibility for the integrity of the data. Due to the multidisciplinary nature of the article and the important role of both corresponding authors in various stages of the research, including design, data analysis, and writing, 2 corresponding authors have been introduced to reflect the equal contribution and importance of each of their expertise.

##  Conflict of Interest

The authors declare that there is no conflict of interest.
